# Supplementary material for: Paracrine Signaling from Breast Cancer Cells Causes Activation of ID4 Expression in Tumor-Associated Macrophages
Source: Cells. 2020 Feb 11;9(2):418. doi: 10.3390/cells9020418 (PMC7072237; doi:10.3390/cells9020418)

Supplementary Figure 1. Images of ID4 and CD68 staining in a representative triple-negative breast cancer case showing high ID4 expression and high leukocyte and macrophage infiltration. Positive expression of ID4 is visible in tumor cells and in cells of the leukocytic infiltrate.

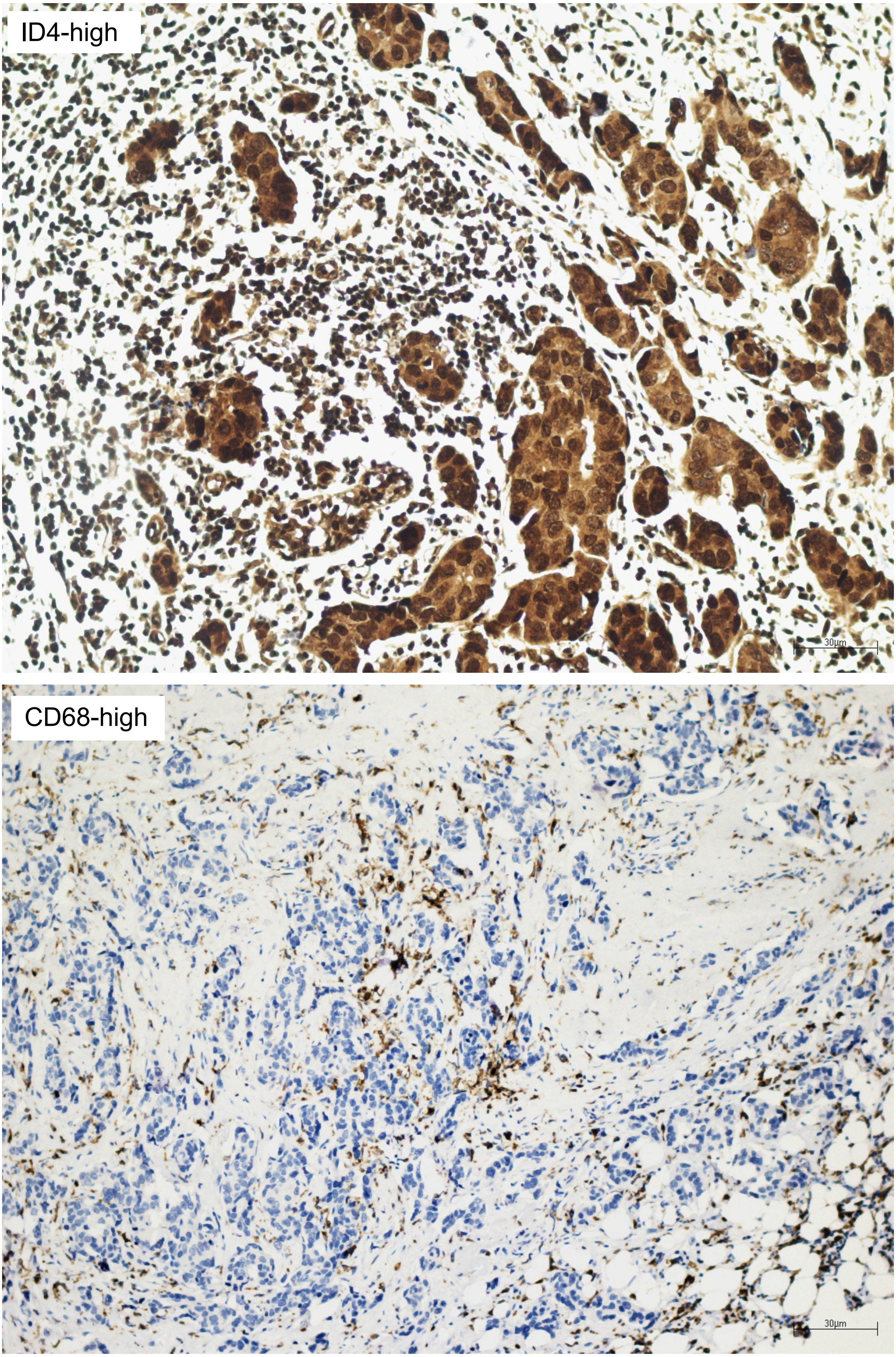

Supplementary Figure 2. A. ID4 mRNA expression in differentiated HL60 cells cultured in RPMI medium or in CM from the indicated breast cancer cells for 24h. **B**. ID4 protein detected by immunocytochemistry (ICC) in differentiated HL60 cells cultured in RPMI medium or in CM from MDA-MB-468 (CM 468) or MDA-MB-231 (CM 231) cells for 24h. **C**. ID4 mRNA expression in differentiated HL60 cells cultured for 24h in CM from MDA-MB-468 cells or in RPMI medium containing the indicated macrophage-activating compounds [LPS 1μg/mL; TNF-alpha 50ng/mL; IL4/IL13 20ng/mL each]. **D-E**. Human fibroblasts were grown in presence of CM from MDA-MB-468 and SKBR3 cells for 24h and analysed for ID4 mRNA (D) and protein (E) expression. **F-G**. ID4 mRNA expression in HL60, Monomac-6 and THP-1 cells cultured in RPMI or in CM from ID4-overexpressing cells (ID4-HA) or control CM (EV). **H**. ChIP analysis of ID4 promoter in RAW264.7 cells cultured for 16h in CM from breast cancer cells, previously transfected with empty vector (CM EV) or with ID4 expression vector (CM ID4), performed using the indicated antibodies. Enrichment of ID4 promoter has been normalized using a negative control genomic region.

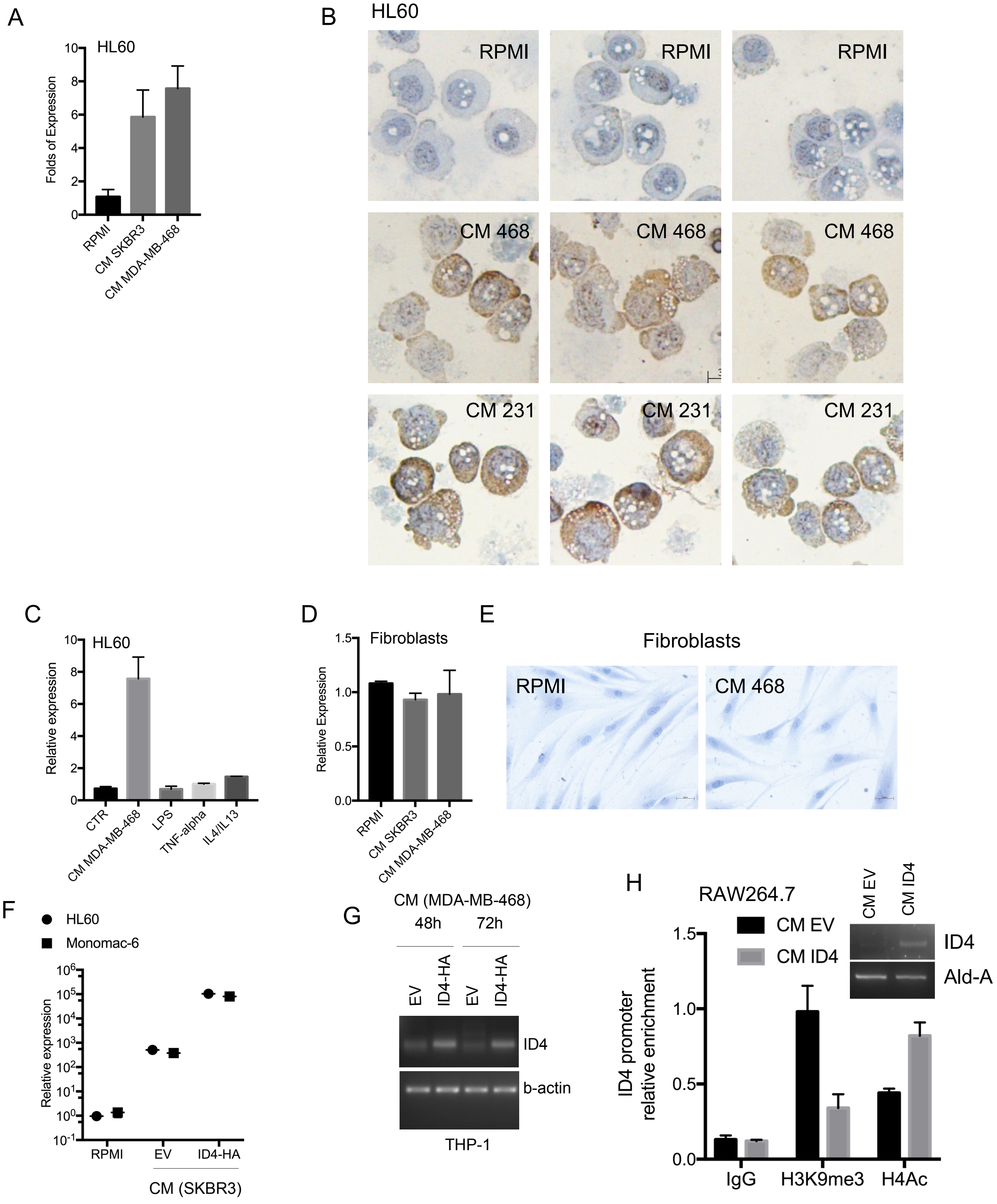

Supplement: Supplementary file 1 [file cells-09-00418-s001.pdf]
